# Medical Institute of Louisville

**Published:** 1845-01

**Authors:** 


### THE WESTERN JOURNAL.

NEW SERIES.—VOL. III.—NO. I.

LOUISVILLE, JANUARY 1, 1845.

### MEDICAL INSTITUTE OF LOUISVILLE.

We have before us, just issued from the press, the Eighth Annual Catalogue of this Institution. It numbers 286, or 24 more than that of any preceding session. 'Till the making up of this class, that of Transylvania University, in 1825–'6, 19 years ago, was the largest ever assembled in the Valley of the Mississippi. By a curious coincidence, it was the Eighth Session of that school which brought a more numerous class than it ever attracted before or since. We are unwilling to believe, that the classes of the Institute are to follow the same law, and not again to equal that of this year. At the time referred to, Transylvania had six professors, of whom one-half are now in the Institute. Their tickets were $20 each, making $120; but the professors received for them the paper of the Commonwealth's Bank, which had depreciated to fifty cents on the dollar; so that attendance on all the lectures cost, in fact, but $60. It was, at the time supposed, that this was one cause of the numerous class of that session. That nothing of the kind has operated to aid the Institute the present session, is obvious, from the fact that it has seven professors, whose tickets amount to $105; and no depreciated bank paper is received, except that of Alabama, which is at a discount of 5 *per cent.* only. It would seem, then, to be the intrinsic advantages—real or supposed—of the Institute, that have secured its present encouraging class. It may be said, however, that the population of the Valley of the Mississippi has greatly increased in 19 years. This is true, but as we shall presently see, the number of schools has increased at a still higher ratio. Before proceeding to enumerate them, we will compare the two catalogues of which we have spoken, and see the relative numbers from different States.

| MEDICAL INSTITUTE OF LOUISVILLE, 1844–'5. | | TRANSYLVANIA UNIVERSITY, 1825–'6. | |
|---|---|---|---|
| Kentucky, | 87 | Kentucky, | 101 |
| Tennessee, | 56 | Tennessee, | 53 |
| Mississippi, | 44 | Mississippi, | 7 |
| Alabama, | 31 | Alabama, | 30 |
| Indiana, | 14 | Indiana, | 5 |
| Missouri, | 13 | Missouri, | 5 |
| Illinois, | 10 | Illinois, | 4 |
| Ohio, | 9 | Ohio, | 8 |
| Arkansas, | 6 | Arkansas, | 1 |
| Louisiana, | 5 | Louisiana, | 3 |
| North Carolina, | 4 | North Carolina, | 13 |
| Georgia, | 4 | Georgia, | 7 |
| Virginia, | 1 | Virginia, | 25 |
| Iowa, | 1 | Iowa, | 0 |
| Texas, | 1 | Texas, | 0 |
| South Carolina, | 0 | South Carolina, | 18 |
| New York, | 0 | New York, | 1 |
| Maryland, | 0 | Maryland, | 1 |
| Total, | 286 | Total, | 282 |

By an examination of these columns, we find, that ours is more of a Western class than that of 1825, the whole except 9 being from the Valley of the Mississippi; while of the class of 1825, 65 were from the old States. We also find, that the increase has been from the States of Mississippi, Indiana, Missouri, Illinois, Arkansas, and Louisiana—most of all from the first. On the other hand, there are 14 fewer from Kentucky, than Transylvania then had; while Tennessee, Alabama, and Ohio, give about the same number as they then gave.

Let us now turn to the relative number of schools at the two periods. We do not recollect precisely when the schools of Charleston, S. C., and Charlottsville, Va., were established, but if previous to 1825, they were then in their infancy, and may be included in the catalogue of creations since that time. To these we may add, the second Virginia school at Richmond, and the school of Georgia. To the establishment of these four Institutions, we may in *part* ascribe our small number of pupils from Virginia, the Carolinas, and Georgia; but it is also partly referrible to the greater distance of Louisville than Lexington, from those States. What number Transylvania has this year, from them, we have not heard. When we direct our attention to the West, we find, as already stated, 277 of our pupils are supplied by *it*, while in 1825 it supplied but 217—difference 60. Now, in 1825, there were but two schools in the West, that of Lexington and that of Cincinnati; but at present there are, in addition, one in New Orleans, two in St. Louis, two in Illinois, one in Indiana, and two in Ohio, making, with the Institute, eleven in all. How can it be, that the Institute has from the West 60 students more than Transylvania had in 1825, when nine new schools have been established since that time? It results from the increase of population; which in the West, from the Lakes to the Gulf, was (exclusive of Michigan) in 1820, 2,203,568, and in 1840, 6,103,161, giving an increase of 277 *per cent.* But during the same period, the number of schools increased 550 *per cent.*, or at a ratio about twice as great. The friends of the Institute may, then, without boasting, or speaking invidiously of its sister institutions, claim that it exerts a powerful influence on the profession in the West, and is decidedly prosperous.

When we receive the catalogues of the various Western schools we shall compare the whole number of pupils, with the population supplying them. D.

